# NBS-LRR Protein Pik-H4 Interacts with OsBIHD1 to Balance Rice Blast Resistance and Growth by Coordinating Ethylene-Brassinosteroid Pathway

**DOI:** 10.3389/fpls.2017.00127

**Published:** 2017-02-06

**Authors:** Hao Liu, Shuangyu Dong, Fengwei Gu, Wei Liu, Guili Yang, Ming Huang, Wuming Xiao, Yongzhu Liu, Tao Guo, Hui Wang, Zhiqiang Chen, Jiafeng Wang

**Affiliations:** National Engineering Research Center of Plant Space Breeding, South China Agricultural UniversityGuangzhou, China

**Keywords:** NBS-LRR resistance gene, homeodomain proteins, ethylenes, brassinosteroid signaling, fungal resistance.

## Abstract

The regulation of innate immunity and plant growth, along with the trade-off between them, affects the defense and recovery mechanisms of the plant after it is attacked by pathogens. Although it is known that hormonal crosstalk plays a major role in regulating interaction of plant growth and PAMP-triggered immunity, the relationship between plant growth and effector-triggered immunity (ETI) remains unclear. In a large-scale yeast two-hybrid screening for Pik-H4-interacting proteins, a homeodomain transcription factor OsBIHD1 was identified, which is previously known to function in biotic and abiotic stress responses. The knockout of *OsBIHD1* in rice lines carrying *Pik-H4* largely compromised the resistance of the rice lines to *Magnaporthe oryzae*, the fungus that causes rice blast. While overexpression of *OsBIHD1* resulted in enhanced expression of the pathogenesis-related (*PR*) and ethylene (ET) synthesis genes. Moreover, OsBIHD1 was also found to directly bind to the promoter region of ethylene-synthesis enzyme OsACO3. In addition, *OsBIHD1* overexpression or deficiency provoked dwarfism and reduced brassinosteroid (BR) insensitivity through repressing the expression of several critical genes involved in BR biosynthesis and BR signaling. During *M. oryzae* infection, transcript levels of the crucial BR catabolic genes (*CYP734A2*, *CYP734A4*, and *CYP734A6*) were significantly up-regulated in *OsBIHD1-OX* plants. Furthermore, OsBIHD1 was found to be capable of binding to the sequence-specific *cis*-elements on the promoters of *CYP734A2* to suppress the plant growth under fungal invasion. Our results collectively suggest a model that OsBIHD1 is required for Pik-H4-mediated blast resistance through modulating the trade-off between resistance and growth by coordinating brassinosteroid-ethylene pathway.

## Introduction

Plant growth and disease resistance have been regarded as two distinct and divergent systems. However, to fend off pathogens, plants must effectively integrate multiple signals including biotic and abiotic stressors to protect themselves from pathogen invasion (Wang and Wang, 2014). Plant defense responses such as pathogen-associated molecular pattern (PAMP)-triggered immunity (PTI) and effector-triggered immunity (ETI) depend upon critical switch, selectively repress growth and focus the energy on resisting pathogen invasion(Liu et al., 2014). The molecular trade-off between the growth and immunity is crucial to the health and survival of plants, which requires integration of the growth and immunity pathways with developmental process (Chandran et al., 2014).

*Pik-H4* is an allele of the major resistance (R) gene *Pi-k* which consists of two adjacent nucleotide-binding domain and leucine-rich repeat (NLR) genes, *Pik_1_-H4* and *Pik_2_-H4* (Xiao et al., 2011). Previous studies have suggested that Pikh-1 directly interacts with the *M. oryzae* effector Avr-Pik and acts as an adaptor to relay signals between Avr-Pik and Pikh-2 (Zhai et al., 2014). The Avr-PikD and Pikp-1 interaction has been recently dissected from the crystal structure (Lu et al., 2010). These studies illustrate the detailed molecular mechanism of an initial recognition event mediated by NLR proteins that integrate an immunity response to rice blast resistance. On the other hand, there is very little evidence for downstream resistance mechanisms induced by the R proteins under *Magnaporthe oryzae* attack. The panicle blast resistance protein Pb1 specifically interacts with WRKY45 to regulate the SA immunity pathway. Pb1 overexpression enhances WRKY45 accumulation and shields it from ubiquitin-mediated proteasomal degradation. Accordingly, WRKY45 is an essential downstream regulator involved in Pb1-dependent blast resistance (Inoue et al., 2013).

In a previous yeast two-hybrid screening for Pik-H4 interacting proteins, we identified a homeodomain-containing protein, OsBIHD1, which has been previously found in suppression subtractive hybridization (SSH) assay for different BTH-responsive cDNA clones (Luo et al., 2005a). Here, we further illustrate the function of the OsBIHD1 in Pik-H4-mediated blast resistance. The results show that OsBIHD1 physically interacts with Pik-H4 and is required for Pik-H4-mediated resistance. During *M. oryzae* invasion, OsBIHD1 regulates blast resistance through direct activation of ET signaling pathway. At the same time, OsBIHD1 suppresses plant growth through directly activating the BR catabiotic genes. This study demonstrates that OsBIHD1, served as a critical molecular switch, coordinates the tradeoff between growth and ETI-triggered immunity in rice.

## Materials and Methods

### Plant Materials and Treatments

*Oryzae sativa japonica* cultivar *Pik-H4 NIL* was used as the wild-type rice strain in this study (Xiao et al., 2011). *Pik-H4 NIL* contains the *Pik-H4* resistance gene (an allele of *Pik* locus) in the susceptible cultivar *LTH* background. The *M. oryzae* race GDYJ7, one of the primary *M. oryzae* races found in Guangdong Province, China, is incompatible with *Pik-H4*.

Eight-week-old rice seedlings grown under natural light in a greenhouse at 26°C were used for inoculation of rice blast fungus. For fungal inoculation, freshly prepared *M. oryzae* spores (1 × 10^5^ conidia/mL 0.02% v/v gelatin) were sprayed onto the rice leaves using an air sprayer. Inoculated plants were kept in a humidity chamber in the dark at 28°C for 24 h, and the plants were then transferred to the normal growth condition. The local lesions were observed 5 days later. The total local lesions area of whole single plant was calculated, and the total area of all the investigated leaves in the whole plant was counted. Differences in blast resistance were determined by the proportion of the lesion area divided by the total leaf area on the same leave. All the experiments were performed in triplicate.

### Yeast Two-Hybrid Assay

Coding sequences of *Pik_1_-H4* and were *Pik_2_-H4* cloned into the BD (binding domain) plasmid pGBKT7 by homologous recombination in yeast strain Y2H gold. Yeast cells containing the resulting constructs BD-Pik_1_-H4 were used as bait to screen for interacting-proteins from a rice yeast two-hybrid cDNA library, according to the manufacturer’s instructions of Clontech yeast two-hybrid handbook. The transformed yeast cells were cultured on SD/-Trp/-Leu and SD/-Trp/-Leu/-His/-Ade+3AT+X-α- GAL plates and results were scored after 3 days incubation at 30°C.

### Bimolecular Fluorescence Complementation (BiFC) Assay

For BiFC assays, the coding regions of *Pik_1_-H4* and *OsBIHD1* were separately cloned into the *Age*I/*Nhe*I sites of BiFC vectors pUC-NE1L2L-nsI and pUC-CE1RL2R-nsI, to generate Pik_1_-H4-nYFP and OsBIHD1-cYFP constructs labeled at their amino and carboxyl termini, respectively (Luo et al., 2013). Rice protoplasts were isolated based on the methods reported by Yang et al. (2014) with slight modifications. Briefly, 50 rice seedlings were cut into approximately 0.5 mm strips, and then incubated in an enzyme solution (1.5% Cellulase RS, 0.75% Macerozyme R-10, 0.6 M mannitol, 10 mM MES at pH 5.7, 10 mM CaCl_2_ and 0.1% BSA) for 4–5 h in the dark with gentle shaking (60–80 rpm). After washing twice with W5 solution (154 mM NaCl, 125 mM CaCl_2_, 5 mM KCl, and 2 mM MES at pH 5.7), the residues were resuspended in MMG solution (0.4 M mannitol, 15 mM MgCl_2_, and 4 mM MES at pH 5.7). The recombinant constructs in pairs were co-transfected into rice protoplasts and the fluorescent signals were examined by confocal microscopy Carl Zeiss LSM780.

### GST Pull Down Assay

The full length *Pik_1_-H4 CC* domain cDNA sequence including stop codon was cloned into the *Bam*HI*/Eco*RI sites of pGEX4p-1 and the *OsBIHD1*_207-527__aa_ cDNA insert was cloned into the *Bam*HI sites of pET28a. Expression of the Pik_1_-H4 CC-GST and OsBIHD1_207-527__aa_-His fusion proteins were induced with 0.5 mM IPTG (isopropyl β-D-thioglucosidase) for 12 h at 37°C in *Escherichia coli* strain BL21.The His-tagged proteins were incubated with purified GST-Pik1-H4 CC or GST alone bound to glutathione beads. After 4 h of incubation at 4°C, the beads were extensively washed four times with GST binding buffer (PBS, pH 7.2). Components bound to the beads were eluted by boiling in SDS sample buffer, and then separated on a SDS-PAGE gel and immune-blotted with anti-His and anti-GST antibodies.

### Total RNA Extraction, Real-Time PCR Analysis of Gene Expression

Total RNA was extracted from 100 mg of fourth-leaf-stage rice seedling with Trizol Reagent (Invitrogen, Beijing, China) and reverse-transcribed using PrimeScript RT reagent Kit (Takara, Dalian, China) according to the manufacturer’s instructions. The cDNA was quantified by real-time PCR using a 20 μl reaction volume and SYBR Premix ExTaq^TM^ (TaKaRa, Dalian, China) on an ABI StepOne Plus system. Primers used for PCR analysis are shown in Supplementary Table S2. Differences in gene expression were expressed as fold change relative to control and were calculated using the 2^-ΔΔCT^ method. Each measurement was carried out in triplicate, and the error bars represent SE of the mean of fold changes for three biological replicates.

### Generation of the *OsBIHD1-OX* and *osbihd1-ko* Transgenic Plants

The full-length of *OsBIHD1* cDNA was isolated by RT-PCR from the leaves of fourth-leaf-stage rice plants using the cDNA F/R primers (Supplementary Table S1) encompassing the translation start and stop codons. This cDNA insert was digested with *BamH*I and cloned between the maize ubiquitin promoter and the Nos terminator in the plant expression vector pOX containing the hygromycin resistance gene as selection maker. CRISPR/Cas9 technology was used to generate *osbihd1-ko* plants. As reported by Ma et al. (2015), a 20 bp DNA fragment including a protospacer-adjacent motif (PAM) of the first exon of *OsBIHD1* nucleotide sequence was fused with a U6a-gRNA box, and the resulting DNA insert digested with *Bsa*I was inserted into the pYLCRISPR/Cas9PUbi-Hi vector. pOX-*OsBIHD1* and pYLCRISPR/Cas9-*OsBIHD1* were then introduced into agrobacterium strain EHA105 and then transformed to wild-type (*Pik-H4 NIL*) calli, as described previously. Transgenic rice plants were regenerated from the transformed calli on selection media containing 50 mg/L hygromycin and 250 mg/L cefotaxime. *OsBIHD1* levels in the transgenic rice plants were further confirmed with target site sequencing and real-time PCR.

### Yeast One-Hybrid Assay

The 2 Kb promoter sequences of *OsACO3* and *CYP734A2* were cloned into the *Eco*RI*/Mlu*I sites of pHIS2 (Clontech) and full length cDNA of *OsBIHD1* was inserted into the *Nde*I*/Eco*RI sites of AD2 (Clontech). The constructs were co-transformed into the yeast strain AH109 (Clontech). The positive transformants were grown on SD/-Trp/-Leu/-His plates containing 100 mM 3-AT and 10 mM X-α-gal for 3 days at 30°C.

### EMSA Assay

DNA binding activity of the recombinant OsBIHD1_207-527aa_ protein was analyzed by an electrophoretic mobility shift assay (EMSA) assay. Labeled probe contained the OsBIHD1 binding site (TGTCA) and the competitor oligonucleotide contained a mutated OsBIHD1 binding motif (TCTCA). Synthesized probe and competitor fragments from the 3′ terminus were annealed and labeled with biotin (Invitrogen). DNA binding reactions were performed at 25°C for 30 min in binding buffer (Chemiluminescent EMSA Kit GS009, Beyotime biotechnology) and subjected to EMSA assay using 10% polyacrylamide gels in 0.5x Tris-borate-EDTA buffer.

### Lamina Joint Test

The lamina joint tests were performed as described by Chen et al. (2015) with slight modifications. Sterilized seeds were germinated in water for 3 days and well-germinated plants were transferred onto 1/2 × Murashige and Skoog medium containing 1% agar and grown for an additional 5 days. Following this, a 500 ng/mL solution of brassinolide (Sigma–Aldrich) in 100% ethanol was applied to the tip of the second leaf blade. Three days later the lamina joint angles of the second leaves were measured.

### Transcriptional Activity Assay in Tobacco Leaves and ChIP-qPCR

The promoter sequences of *OsACO3* and *CYP734A2* were cloned into the *Bam*HI/*Nco*I sites of pCAMBIA1305, the last constructs were co-transformed into tobacco leaves with 35S:OsBIHD1 induced by Agrobacterium (strain: EHA105). The tobacco leaves were incubated in a solution containing 50 mM NaPO_4_ buffer (pH 7.0), 5 mM K_3_Fe(CN)_6_, 5 mM K_4_Fe(CN)_6_, 0.1% Triton X-100, and 1 mM X-Gluc at 37°C. CHIP-qPCR was performed as described previously (Yang et al., 2014) Rice protoplast isolated from 200 rice seedlings that were cut into approximately 0.5 mm strips, and then incubated in an enzyme solution (1.5% Cellulase RS, 0.75% Macerozyme R-10, 0.6 M mannitol, 10 mM MES at pH 5.7, 10 mM CaCl_2_ and 0.1% BSA) for 4–5 h in the dark with gentle shaking (60–80 rpm). OsBIHD1-GFP and empty GFP were transiently co-expressed in rice protoplasts by 40% PEG induction. Then, harvested and extracted the total protoplast chromatin. The chromatin preparations were sonicated into 0.2–0.5 kb fragments. Specific antibodies against GFP (Cat.No.11814460001, Roche) were added to the chromatin solution, which was precleared with salmon sperm DNA/Protein *A*-agarose beads. The precipitates were eluted from the beads. Cross-links were reversed, and residual proteins were removed by incubation with proteinase K. DNA was recovered using the QIAquick spin column (Qiagen, Valencia, CA, USA). Quantitative PCR was used to determine the amounts of genomic DNA enriched in the chromatin samples. The primers were designed to amplify DNA fragments of 150–250 bp (Yun et al., 2012).

## Results

### Pik-H4 Interacts with OsBIHD1 through Its CC Domain

**FIGURE 1:**
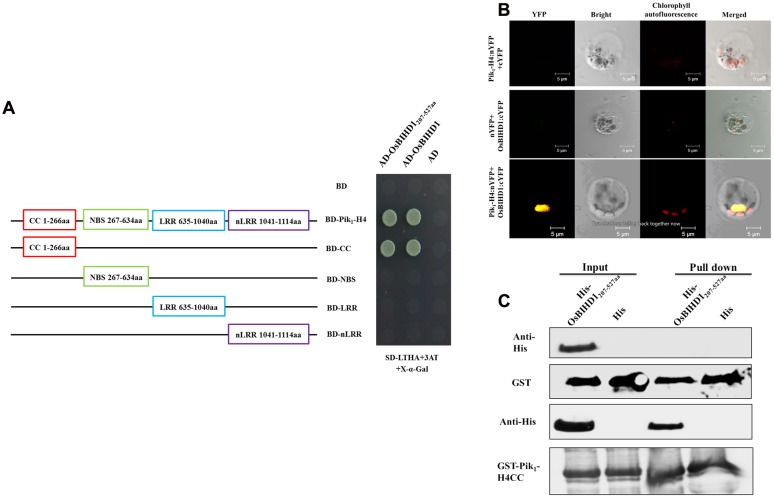
**Interaction between Pik_1_-H4 and OsBIHD1. (A)** Yeast two-hybrid analysis to confirm the specificity of the interaction between Pik_1_-H4 and OsBIHD1. CC domain (1–266 aa), NBS domain (367–634 aa), LRR domain (635–1040 aa) and nLRR domain (1041–1114 aa) of Pik1-H4 were fused to GAL4 DNA-binding domain, respectively, and expressed in combination with truncation construct of OsBHD1 (207–527 aa) fused to the GAL4 activation domain (AD) in yeast strain Y2H Gold. **(B)** Bimolecular fluorescence complementation (BiFC) analysis of Pik_1_-H4-OsBIHD1 interaction in rice protoplasts. cDNA coding for the full length Pik1-H4 protein was fused to the N-terminal half of YFP and co-transformed in rice protoplasts together with the cDNA coding for OsBIHD1 fused to the C-terminal moiety of YFP. The yellow fluorescence (YFP), a bright field image and chlorophyll autofluorescence (Chl) were recorded and the resulting images were merged. Scale bar is 5 μm. **(C)** *In vitro* GST pull-down assay between the Pik_1_-H4 CC and OsBIHD1_207-527__aa_. Hexahistidine-tagged OsBIHD1_207-527aa_ (His-OsBIHD1_207-527aa_) and GST-fused Pik_1_-H4_1-266aa_ (GST-Pik_1_-H4 CC) were expressed in *Escherichia coli* and used for the analysis.

To investigate rice proteins interacting with Pik_1_-H4 (one protein of the resistance protein pair Pik_1_-H4/Pik_2_-H4), we previously performed a yeast two-hybrid screen using a rice cDNA library and identified a homeodomain transcription factor OsBIHD1. The HD domain (207–527 aa) of OsBIHD1 was sufficient for binding to Pik_1_-H4 (**Figure 1A**), while this domain didn’t show any interacting with Pik_2_-H4 (Supplementary Figure S1). To confirm the specificity of this interaction, we sought to identify the OsBIHD1-interacting sites in the Pik_1_-H4 molecule which consists of four domains: CC (coiled-coil: 1–266 aa), NBS (nucleotide-binding: 267–634 aa), LRR (Leucine-rich repeats: 635–1040 aa), non-LRR domain (carboxyl-terminus: 1041–1114 aa). The strongest binding to OsBIHD1 or HD domain was achieved with the full-length Pik_1_-H4 and CC domain, whereas the NBS, LRR and non-LRR domains did not show any interaction (**Figure 1A**).

To confirm our yeast two-hybrid results, we used bimolecular fluorescence complementation (BiFC) assay to test the interaction between Pik_1_-H4 and OsBIHD1 in rice protoplast cells. Rice protoplast cells that were co-transfected with the vectors expressing Pik_1_-H4:nYFP and OsBIHD1:cYFP displayed YFP fluorescence under laser confocal scanning microscopy (**Figure 1B**). Further subcellular localization assay showed that Pik_1_-H4 and OsBIHD1 co-localized in the nucleus (Supplementary Figure S2A), indicating that Pik_1_-H4 interacts with OsBIHD1 in the nucleus. Direct binding was also observed between recombinant Pik_1_-H4 CC and OsBIHD1 HD *in vitro* in a GST pull-down assay (**Figure 1C**). Taken together, these results confirm a direct interaction between Pik_1_-H4 and OsBIHD1, especially between Pik_1_-H4 CC domain and OsBIHD1 HD domain, and the interaction may happen in the nucleus of rice cells.

To determine whether the OsBIHD1 HD region was sufficient for transcriptional activation, we tested three truncated mutants of OsBIHD1. The transcriptional activity assay indicated that the HD domain was not active in this assay whereas the full-length protein was (Supplementary Figure S2B). This result indicates that the HD region is responsible for interacting with Pik_1_-H4 and the activity domain is contained within OsBIHD1_1-180__aa_.

### *Pik-H4* Mediated Blast Resistance Depends on *OsBIHD1*

We firstly examined the expression pattern of *OsBIHD1* over a time course of 72 h after inoculation with *M. oryzae* by quantitative RT-PCR (qRT-PCR). The *OsBIHD1* expression at the mRNA level was significantly increased at 6 h and reached its lowest level at 36 h, and then it was decreased at a relatively low level from 60 to 72 h after inoculation in wild-type plants. In addition, the transcript abundances of *Pik_1_-H4* and *Pik_2_-H4* also were up-regulated over a time course of 48 h after inoculation of blast fungus (Supplementary Figure S3).

**FIGURE 2:**
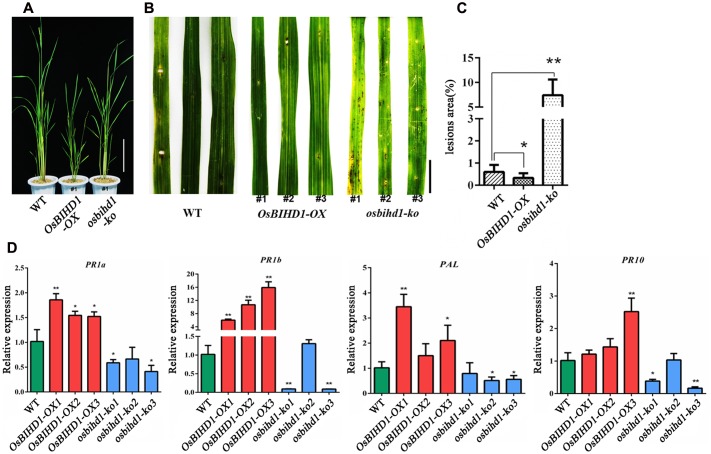
***Pik_1_-H4*-mediated blast resistance requires *OsBIHD1*. (A)** Phenotype of Wild-type and *OsBIHD1* transgenic plants. Scale bar is 20 cm. **(B)** Photographs of blast fungus-inoculated fourth leaves of *OsBIHD1* transgenic plants and wild-type at the six-leaf stage. A conidial suspension of blast fungus race GDYJ7 was sprayed on the leaf surfaces, and local lesions were observed 5 days later. Bar is 2 cm. **(C)** Lesion and total leaf areas measured in 10 independent plants. The indicated values show the ratio of lesion area to leaf area mean ± SD. Asterisks indicate a significant difference according to the *t*-test (^∗^*P* < 0.05, ^∗∗^*P* < 0.01) compared with wild-type. **(D)** Relative expression of pathogen-related genes (*PR1a*, *PR1b*, *PR10*, and *PAL*) in wild-type, *OsBIHD1-OX*, and *osbihd1-ko* plants. Values are mean ± SD of three biological replicates, and asterisks indicate a significant difference according to the *t*-test (*P* < 0.05) compared with WT.

To understand the function of *OsBIHD1*, we introduced *OsBIHD1* overexpression (*OsBIHD1-OX)* and *OsBIHD1* knock-out (*osbihd1-ko*) constructs, respectively, into wild type carrying *Pik-H4*, generating the rice lines *Pik-H4*+/*OsBIHD1*-*OX* and *Pik-H4*+/*osbihd1-ko*, respectively (Supplementary Figure S4 and **Figure 2A**). The expression levels of *Pik_1_-H4* and *Pik_2_-H4* (resistance gene cluster *Pik_1_-H4*/*Pik_2_-H4*) in *Pik-H4*+/*OsBIHD1*-*OX* and *Pik-H4*+/*osbihd1-ko* rice lines were similar to those in the wild-type lines (Supplementary Figure S5), indicating that the *OsBIHD1* overexpression or knock-out did not affect *Pik_1_-H4* expression in these transformants. A blast resistance test with *M. oryzae* race GDYJ7 (carrying *Avr-PikH4*) showed that the resistance was compromised in *osbihd1-ko* plants (**Figures 2B,C**), in which *OsBIHD1* transcript levels in leaves were very low (Supplementary Figure S4), while the levels of resistance in *Pik-H4*+/*OsBIHD1*-*OX* plants were significantly increased compared with the wild type plants (**Figure 2C**).

Previous studies have shown that expression of *OsBIHD1* was activated on treatment with benzothiadiazole (BTH) and OsBIHD1 overexpression resulted in an elevated level of defense-related *PR-1*gene expression in tobacco leaves (Luo et al., 2005b). To further test whether *OsBIHD1* is involved in induction or accumulation of PR-protein mRNAs in rice, the transcript levels of four PR genes: *PR1a*, *PR1b*, *PR10*, and *PAL*, were investigated in *OsBIHD1-OX* and *osbihd1-ko* plants using qRT-PCR. The results showed that the expression of all these genes was down-regulated in *osbihd1-ko* lines in comparison with wild-type plants under normal growth condition, while that of all these genes were significantly up-regulated in *OsBIHD1-OX* lines (**Figure 2D**). These results indicate that *OsBIHD1* is required Pik-H4-mediated blast resistance and acts as a positive regulator downstream of defense signaling transduction through affecting the expression of *PR* genes.

### *OsBIHD1* Activates the ET-Dependent Defense Pathway

**FIGURE 3:**
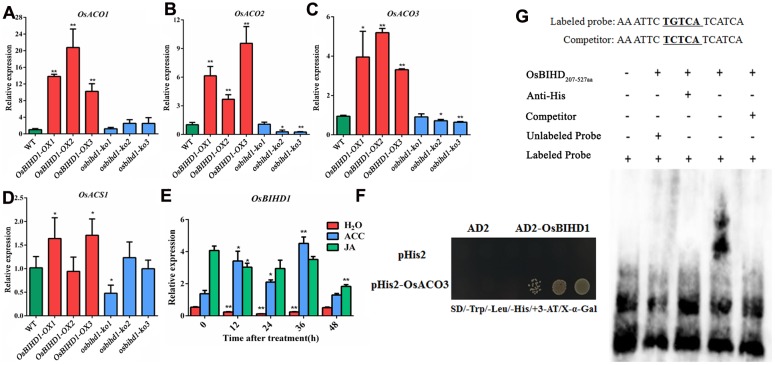
***OsBIHD1* up-regulates ET synthetic gene expression and directly binds the *OsACO3* promoter sequence. (A–D)** Quantitative real-time PCR analyses of *OsACS1*, *OsACO1*, *OsACO2*, and *OsACO3* transcripts in WT and *OsBIHD1* transgenic plants, and asterisks indicate a significant difference according to the *t*-test (*P* < 0.05) compared with WT. **(E)** *OsBIHD1* expression induced by exogenous ACC (1 mM) after 48 h.Sterile H_2_O and exogenous JA (100 μM) were used as controls. Values shown are means ± SD from three independent experiments, and asterisks indicate a significant difference according to the *t*-test (*P* < 0.05) compared with 0 h. **(F)** OsBIHD1 binds the *OsACO3* promoter sequence *in vivo*. Yeast one-hybrid assay results using an X-Gal assay. **(G)** EMSA assay illustrating that the OsBIHD1_207-527__aa_ protein binds to the promoter *cis*-element TGTCA of *OsACO3 in vitro*.

Accumulating evidences have illustrated that exogenous hormones such as SA, JA, and ET precursor 1-amino-cyclopropane-1-carboxylic acid (ACC) are involved in inducing the expression *PR* genes (Takeuchi et al., 2011). It is possible that *OsBIHD1* may regulate the expression of *PR* genes through affecting the hormone-regulated pathway. The expression of a subset of key genes involved in ET biosynthesis including *ACO1* (Iwamoto et al., 2010), *ACO2* (Chae et al., 2000), *ACO3* (Iwai et al., 2006), and *ACS1* (Iwai et al., 2006) were analyzed with qRT-PCR in *Pik-H4+/OsBIHD1-OX* and *Pik-H4+/osbihd-ko* transgenic plants. The results showed that the transcript levels of *ACO* family genes were all up-regulated in *OsBIHD1-OX* plants while that of the *ACS* family gene were only slightly alerted (**Figures 3A–D**; Supplementary Figure S6). In addition, *OsBIHD1* expression could be induced by exogenous application of ACC (**Figure 3E**). These results suggest that *OsBIHD1* is most likely involved in ET-mediated immunity.

*OsBIHD1* encodes a homeodomain protein with DNA binding activity and directly binds to TGTCA motif in the *cis*-element sequence. To identify whether the motif is present in the promoters of the ET biosynthesis genes, we used the plant *cis*-acting regulatory DNA elements (PLACE) database (Higo et al., 1999) and found there were many potential OsBIHD1 binding sites in the promoter region of *OsACO3* (Supplementary Figure S7). To further elucidate whether OsBIHD1 directly activated the expression of *OsACO3*, yeast one-hybrid assay and an EMSA were carried out. Our results showed that OsBIHD1 physically bound to the *cis*-acting elements of *OsACO3 in vivo* (**Figure 3F**) and OsBIHD1 protein caused a mobility shift in the labeled probes from the *cis*-acting elements of *OsACO3*, which migrated more slowly than the free probes (**Figure 3G**). These results demonstrate that OsBIHD1 is involved in activating the ET-dependent defense pathway through regulating *OsAOC3’s* expression by directly binding to the *OsAOC3* promoter region.

### Overexpression of *OsBIHD1* Leads to BR Insensitivity

**FIGURE 4:**
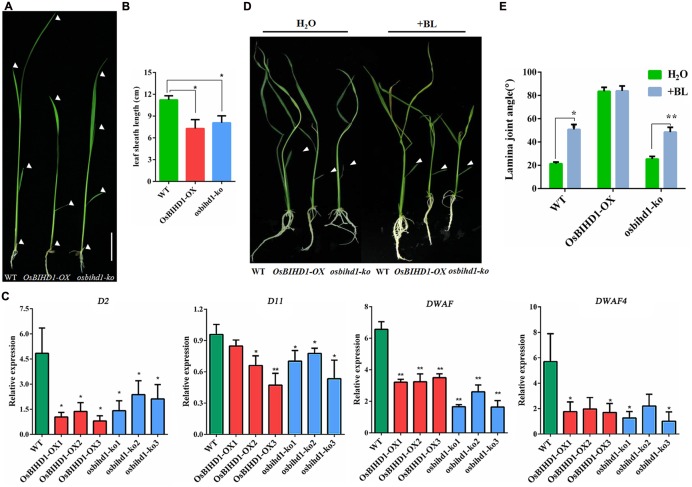
***OsBIHD1* alters the expression of BR biosynthesis genes. (A)** Phenotype of *OsBIHD1* transgenic plants and wild-type seedlings. Scale bar is 4 cm. **(B)** Quantification of fourth leaf sheath length (*n* = 10). Values shown are means ± SD, and asterisks indicate a significant difference according to the *t*-test (^∗^*P* < 0.05, ^∗∗^*P* < 0.01) compared with wild-type. **(C)** Relative expression of BR biosynthesis genes *D2*, *D11*, *DWARF4*, and *DWARF* in wild-type, *OsBIHD1-OX*, *osbihd1-ko*. Values shown are means ± SD with three independent replicates, and asterisks indicate a significant difference according to the *t*-test (*P* < 0.05) compared with WT. **(D)** Phenotypes of wild type, *OsBIHD1-OX*, and *osbihd1-ko* plants after BR treatment. Twelve days old plants were treated with 2 μl BL (500 ng/L) by the micro-drop method. Controls received H_2_O. BL is brassinolide. **(E)** Quantification of lamina joint angle of second leaves (*n* = 10). Values shown are means ± SD, and asterisks indicate a significant difference according to the *t*-test (^∗^*P* < 0.05, ^∗∗^*P* < 0.01) compared with control group.

Homeodomain-containing proteins are involved in BR phytohormone signaling transduction through activation of BR biosynthesis or catabolism in rice (Ito et al., 2002; Tsuda and Hake, 2015). In this study, both *OsBIHD1* overexpression and knock-out has obvious effects on plant seedlings such as dwarfing, increasing lamina joint angles and erect leaves (**Figures 4A,B**; Supplementary Figure S4). In order to investigate the possible roles of *OsBIHD1* in BR biosynthesis, the expression of BR biosynthesis genes *D2* (Hong et al., 2003), *D11* (Tanabe et al., 2005), *DWARF* (Hong et al., 2002), and *DWARF4* (Sakamoto et al., 2006) were analyzed with qRT-PCR. The results showed overexpression of *OsBIHD1* did not result in any obvious up-regulation of BR biosynthetic genes, indicating that *OsBIHD1* is not associated with BR biosynthesis (**Figure 4C**).

Brassinosteroid plays important roles in plant growth and development and particularly in leaf morphology (Saini et al., 2015). Considering the increase in leaf joint angle and the erect phenotype in the *OsBIHD1* transgenic lines, we suspected that *OsBIHD1* overexpression might lead to BR insensitivity. We then performed a lamina joint test for BR sensitivity as described previously (Chen et al., 2015). When wild type and *osbihd1-ko* seedlings were treated with brassinolide (500 ng/L), their lamina joint angles greatly increased (**Figures 4D,E**). By contrast, the angle of *OsBIHD1-OX* plants barely increased and the leaf blades were kept erect. Therefore, *OsBIHD1* overexpression resulted in BR insensitivity, suggesting that OsBIHD1 suppresses the BR pathway by repressing BR signaling or catabolism, but not by suppressing BR biosynthesis. In addition, we measured transcript levels of BR signaling genes in *OsBIHD1* transgenic plants and wild-type (Supplementary Figure S8), but we could not find clear evidence to explain whether or not *OsBIHD1* modulates the expression of BR signaling-related genes to regulate leaf morphology.

### *OsBIHD1* Positively Regulates the Expression of BR Catabolic Genes to Coordinate Growth-Resistance Crosstalk

**FIGURE 5:**
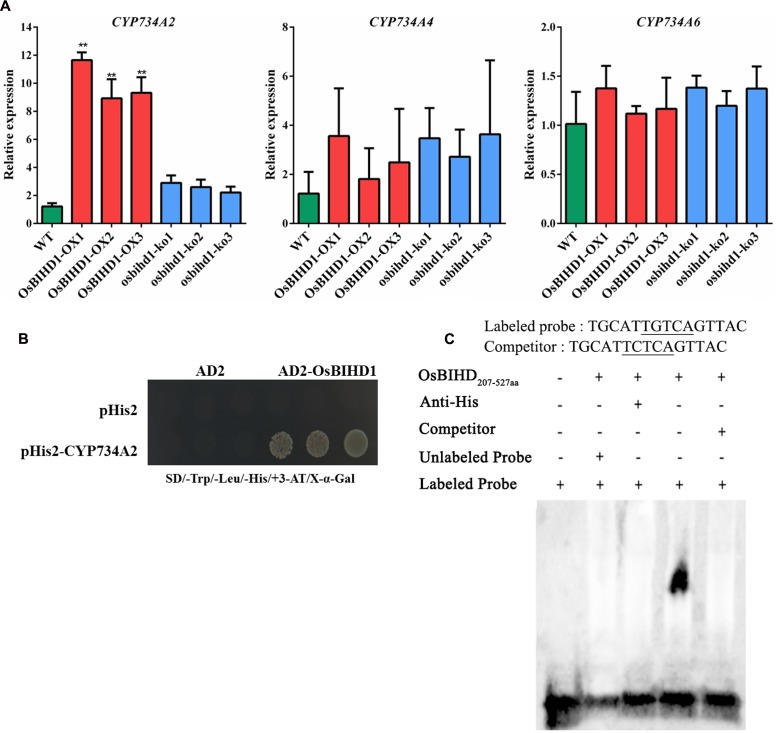
**OsBIHD1 binds to the promoter of the BR catabolic gene *CYP734A2*. (A)** Relative expression of BR catabolism genes in wild-type, *OsBIHD1-OX*, and *osbihd1-ko* plants. Values shown are means ± SD from three independent replicates, and asterisks indicate a significant difference according to the *t*-test (*P* < 0.05) compared with WT. **(B)** Yeast one-hybrid assay identifies OsBIHD1 binding the *CYP734A2* promoter sequence *in vivo.* Reactions were score using an X-Gal assay. **(C)** EMSA analysis of recombinant OsBIHD1_207-527__aa_ protein binding to the promoter *cis*-element TGTCA of *CYP734A2 in vitro*.

The brassinosteroid-deficient phenotypes of *OsBIHD1* transgenicplants might be caused by the activation of BR catabolic genes, which would lead to an increase in the endogenous level of bioactive BR and a more rapid transformation into an inactivate form of BR (Yang et al., 2014). Therefore, the expression of the BR catabolic genes *CYP734A2*, *CYP734A4*, and *CYP734A6* (Sakamoto et al., 2011) was investigated in *OsBIHD1* transgenic plants using qRT-PCR. The results showed the transcript level of *CYP734A4* was only slightly enhanced in both *OsBIHD1-OX* and *osbihd1-ko* lines and that of *CYP734A6* was also not significantly altered (**Figure 5A**). However, *CYP734A2* expression was significantly increased in *OsBIHD1-OX* plants, which might result in a decrease in bioactive BR levels (**Figure 5A**). As a consequence, the accumulation of *CYP734A2* displayed dwarfing and abnormal leaf morphologies. To further determine whether OsBIHD1 activates *CYP734A2* expression, a yeast one-hybrid assay and an EMSA were performed. The results showed OsBIHD1 directly bound to *CYP734A2* promoter *in vitro* and *in vitro* (**Figures 5B,C**; Supplementary Figure S7). Our results revealed that OsBIHD1 promotes the expression of BR catabolic gene *CYP734A2* through directly binding to *CYP734A2*’s promoter region.

### *OsBIHD1* Activates the Hormone-Related Genes Expression

**FIGURE 6:**
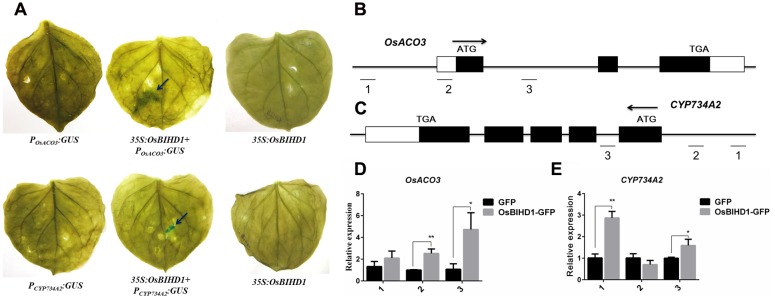
**OsBIHD1 activates the hormone-related genes expression. (A)** OsBIHD1 activated the GUS expression when co-transformed with *OsACO3* and *CYP734A2* promoter sequence into the tobacco leaves. **(B–E)** Results of chromatin immunoprecipitation (ChIP) assays at *OsACO3* and *CYP734A2* loci in rice protoplast system. ChIP analyses at *OsACO3* and *CYP734A2* chromatin regions were performed using antibodies against GFP in rice protoplast system. The reference gene *Ubq13* was used as an internal standard for normalization. Values shown are means ± standard deviation from three parallel biological replicates, and asterisks indicate a significant difference according to the *t*-test (*P* < 0.05) compared with control group.

In spite of the directly bindings between the OsBIHD1 and hormone-related genes have been proved, but the concerned question is whether the OsBIHD1 activates the *OsACO3* and *CYP734A2* expression. To overcome this issue, we thought the transcriptional activity assay could make it convinced that OsBIHD1 activates the transcription of *OsACO3* and *CYP734A2*. Therefore, the last constructs Promoter*_OsACO3_*:GUS and Promoter*_CY P734A2_*:GUS (contain the TGTCA *cis*-element) were co-transformed into tobacco leaves with 35S:*OsBIHD1*, respectively (Supplementary Figure S9). The results of GUS staining showed that OsBIHD1 was able to activate the GUS expression when co-transformed with the *OsACO3* and *CYP734A2* promoters region (**Figure 6A**). Moreover, the results obtained from ChIP-qPCR also proved the same conclusion in rice protoplast system (**Figures 6B–E**). Taken together, we concluded that the OsBIHD1 received the upstream signaling transduction from the Pik_1_-H4, and then directly binding the hormone-related genes promoter region and activating their expression.

### *Pik-H4* Slightly Alters *OsBIHD1* Expression

**FIGURE 7:**
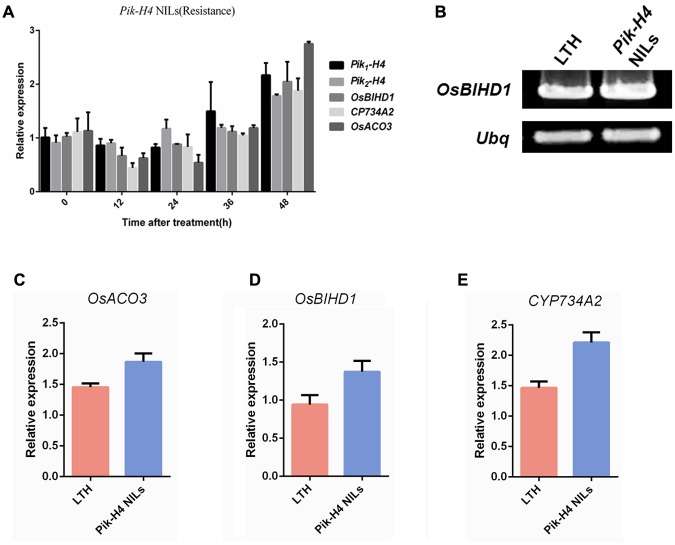
***Pik-H4* positively regulates *OsBIHD1* expression. (A)** Relative expression of *Pik-H4*, *OsBIHD1*, *CYP734A2*, and *OsACO3* over a time course of 48 h after inoculation with *Moryzae oryzae* GDYJ8 in *Pik-H4* NILs. **(B–E)** Relative expression of *OsACO3*, *OsBIHD1*, and *CYP734A2* in *Pik-H4* NILs and LTH plants. Values shown are means ± standard deviation from three parallel biological replicates.

We showed that OsBIHD1 specifically interacted with Pik1-H4, and adjusted the ethylene and BR hormone pathway. But it needed to make it clear that whether the OsBIHD1-mediated transcriptional regulation is Pik-H4 activation dependent. So a *Pik-H4* comprised *M. oryzae* race GDYJ8 (carrying the *Avr-Pita*) was used in this experiment. The results showed that the expression of *Pik1-H4* and *OsBIHD1* was decreased over 24 h after inoculated with GDYJ8 (**Figure 7A**), which was different from that inoculated with GDYJ7 (carrying the *Avr-PikH4*). In additional, we further detected the transcription levels of *OsBIHD1* and hormone-related genes in *Pik-H4* NILs and *Pik-H4* null background rice (**Figures 7B–E**). Totally, these results indicated that *Pik-H4* slightly up-regulates the expression of *OsBIHD1*, ET- and BR- related genes, but the values didn’t exhibit significantly difference between the *Pik-H4* NILs and susceptible variety LTH.

Since of the expression of *OsBIHD1* could be up-regulated after inoculation with *M. oryzae* in BTH-treatment seedlings (Luo et al., 2005a), expression of BR catabolic genes should also be induced with challenge by *M. oryzae*. As we expected, the transcript levels of all BR catabolic genes were significantly up-regulated and peaked in wild type leaves at 36 h, and decreased in *osbihd1-ko* leaves after *M. oryzae* treatment (Supplementary Figure S10), indicating BR catabolic genes depend upon OsBIHD1 to activate their expression to suppress plant growth under pathogen invasion.

## Discussion

**FIGURE 8:**
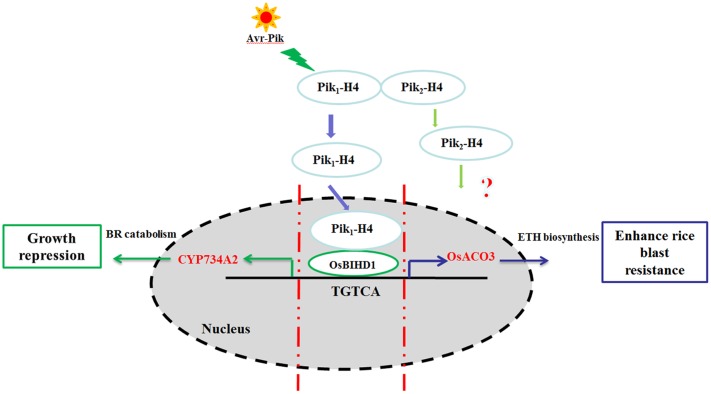
**Model of the Pik-H4 interact OsBIHD1 to regulate rice blast and growth.** The signaling initiating from the *M. oryzae* elicitor Avr-Pik is recognized by the Pik_1_-H4 CC domain and the nuclear Pik_1_-H4 interacts with the OsBIHD1 homeodomain. After signaling from Pik_1_-H4, OsBIHD1 binds the *cis*-element of the *OsACO3* promoter and positively stimulates rice blast resistance *via* the ET defense pathway *OsBIHD1* overexpression has an inhibitory effect on plant growth which promotes the expression of the BR catabolicgene *CYP734A2*. This results in a decrease in bioactive BR. As a consequence, *OsBIHD1* mediates an important trade-off between rice blast resistance and growth.

In a previous yeast two-hybrid screening for Pik-H4 binding proteins, we identified a homeodomain (HD)-containing protein, OsBIHD1, which may be positively involved in activating expression of the defense-related genes in disease resistance responses (Luo et al., 2005b). We showed in this study that the NBS-LRR protein Pik-H4 interacts with OsBIHD1 and the blast resistance by Pik-H4 depends on OsBIHD1. Importantly, OsBIHD1 directly activates ET pathway and positively regulates the expression of BR catabolic genes to coordinate growth-resistance crosstalk. Therefore, OsBIHD1 is a key regulator for the crosstalk between growth and resistance (**Figure 8**).

### Pik-H4 Interacts with OsBIHD1 to Regulate Blast Resistance

The majority of plant *R* genes encode nucleotide-binding leucine-rich repeat (NBS-LRR) proteins that mediate recognition of diverse effectors (Avirulence proteins, Avr). The NBS domain (also called the NB, NB-ARC and Nod domain) is involved in signaling by activation of a kinase or other proteins in the resistance response (DeYoung and Innes, 2006; Ting et al., 2008). LRR domain is thought to be the primary determinant of pathogen recognition specificity or downstream events (Belkhadir et al., 2004). In Plant NBS-LRR proteins, there is a toll/interleukin-1 receptor (TIR) domain or a coiled coil domain (CC), which influences the requirement for distinct downstream signaling components or direct recognition with pathogen effectors (Jia et al., 2000). Currently, more than 24 major R genes that confer resistance against *M. oryzae* in rice have been identified including *Pi-ta* (Bryan et al., 2000), *Pi-k* (Zhai et al., 2011), and *Pb1* (Hayashi et al., 2010), the downstream signaling activated by the recognition of R-Avr remains little known. In rice, the important roles of the transcription factors in R gene-mediated disease resistance have been broadly reported, such as the blast resistance of Pb1 depends on its interaction with a transcription factor WRKY45 (Inoue et al., 2013), and the pattern recognition receptor Xa21 interacts with WRKY62 to regulate *Xoo* resistance (Peng et al., 2008; Park and Ronald, 2012). In this study we showed that Pik-H4 physically interacts with HD transcription factor OsBIHD1 through its CC domain in the nucleus. Knockout of *OsBIHD1* gene down-regulates the expression levels of four PR genes, including *PR1a*, *PR1b*, *PR10*, and *PAL*, which are tightly correlated with the onset of defense responses against a variety of fungal, viral, and bacterial pathogens (Riviere et al., 2008; Zhang and Wang, 2013; Huang et al., 2016). Thus, the blast resistance of *Pik-H4* was also reduced in *osbihd1-ko* mutants. These results indicated *OsBIHD1* is required for *Pik-H4*-mediated blast resistance through protein–protein interaction and *OsBIHD1* is a positive regulator of immunity.

### *OsBIHD1* Directly Activates the ET Pathway to Defend against *M. oryzae*

Effector-triggered immunity is usually accompanied by rapid ET production and a programmed cell death at the site of infection to prevent further infection by the invading pathogens (Yang C. et al., 2015; Zdarska et al., 2015). In *Arabidopsis*, avrRpt2 (effector)-triggered response caused higher ethylene production, which is dependent on RPS2, the R protein corresponding to avrRpt2 (Kunkel et al., 1993; Guan et al., 2015). Higher level of ET production indicates ET signaling pathway may be activated during plant-pathogen interaction through unknown ways. Besides ET, several other plant hormones such as JA, SA, and ABA are also involved in this defense. Blast resistance by Pb1 partially depends on the SA signaling pathway, which is mainly regulated bythe Pb1-interacting transcription factor, WRKY45 (Inoue et al., 2013). Overexpression of *OsBIHD1* led to elevation of the expression of ET biosynthesis *ACO* family genes, suggesting that *OsBIHD1* plays important roles in ET signaling pathway, which is consistent with the evidence that ET is accompanied by elevation of PR genes expression (Yang Y.X. et al., 2015). Yeast one-hybrid and EMSA confirmed that OsBIHD1 directly bounds to TGTCA motif in the *cis*-element sequence of *OsACO3*, indicating that resistance gene *Pik-H4* may depend on *OsBIHD1* to directly regulate the ET defense pathway.

### *OsBIHD1* Plays a Critical Role in the Crosstalk between Plant Growth and Immunity

Previous studies showed that rice KNOX-HD OSH1 proteins are involved in the determination of the state of the shoot apical meristem (SAM) (Tsuda et al., 2014). OSH1 represses the BR phytohormone pathway through activation of BR catabolism genes. These findings provided the evidence that HD containing protein, OsBIHD1, may participate in the regulation of BR phytohormone pathway. Our results demonstrated that*OsBIHD1* overexpression showed an increased lamina joint angle and erect leaves, and this phenotype is in accordance with the BR-deficient and -insensitive rice mutants (Sato et al., 1999).

We next test the expression of the BR catabolic genes *CYP734A2*, *CYP734A4*, and *CYP734A6* in *OsBIHD1* transgenic plants. *CYP734A2* expression was significantly increased in *OsBIHD1-OX* plants and it also was induced by infection with *M. oryzae*, in consistent with *OsBIHD1* expression in *M. oryzae* treatment seedlings (Sakamoto et al., 2011), suggesting the expression of *CYP734A2* is regulated by *OsBIHD1*. Further yeast one-hybrid assay and EMSA showed OsBIHD1 directly bound to *CYP734A2*’s promoter region *in vivo*. These results suggest *OsBIHD1* possibly plays a key role in the crosstalk between plant growth and immunity.

The crosstalk between the BR and PTI immunity clearly revealed that BR signaling transcription factor HBI1 represses the immunity response against *Pseudomonas syringae* in *Arabidopsis* (Fan et al., 2014). Our findings proved Pik-H4 interacts with OsBIHD1 to modulate BR catabolism and plant resistance so that the plants can focus its energy to fend off the pathogen invasion (**Figure 8**). Therefore, we conclude that *OsBIHD1* is most likely *HBI1* to coordinate growth-resistance crosstalk. Whether *OsBIHD1* was involved in the PTI immunity response remains unknown. Though the BR-activated transcription factor BZR1 directly regulates many defense-related genes, *BZR1* itself is not affected by PAMP signaling (Guo et al., 2013; Lozano-Duran et al., 2013). Further characterization of whether OsBIHD1 interacts with BZR1 and HBI1 and the OsBIHD1-interacting network are required to be demonstrated, which will be of great importance for understanding the trade-off between growth and immunity.

However, OsBIHD1 knock-out transgenic plants also showed dwarf and large lamina joint angle phenotype. We suspect that the putative roles of OsBIHD1 are similar to the dwarfism gene *d6*. Loss of function mutant of *D6* in rice exhibited defects in internode elongation and repression in SAM formation (Sato et al., 1999; Nagasaki et al., 2001). In addition, our results suggested that the abnormal phenotype caused by the *OsBIHD1* deficient in rice was attributed to the endogenous BR disorder. Whether the OsBIHD1 involves in other hormones pathway to regulate the plant growth, such as homeodomain containing proteins induce cytokinin (CK) biosynthesis and directly suppress gibberellin (GA) biosynthesis should be illustrated in future work (Jasinski et al., 2005; Yanai et al., 2005).

### OsBIHD1 May Be Modified by Phosphorylation and Ubiquitination

Because *OsBIHD1* overexpression caused abnormal plant phenotypes, plants require *OsBIHD1* expression at a modest level to maintain growth under normal conditions. Once the pathogen has infected, the elicitor AvrPik was recognized by NLR protein Pik-H4 and the conserved CC domain of Pik-H4 maintained an interaction with OsBIHD1 in the nucleus. We do not know whether Pik_1_-H4 contains another domain (LRR domain) responsible for a direct phosphorylation of OsBIHD1 or whether this is dependent upon the CC domain. We suspect that phosphorylated OsBIHD1 further activates the expression of a battery of target genes, including the ET biosynthetic gene *OsACO3* and the BR catabolic gene *CYP734A2*.

We wondered whether *OsBIHD1* was involved in the PTI immunity response and induced crosstalk between immunity and plant growth pathways. If this was the case there would by other transcription factors that regulate *OsBIHD1* expression after the PTI immunity activated by *M. oryzae*. At the conclusion of the immunity response, plants gradually recover from the conflict with *M. oryzae*. Thus, growth prevailed during the remainder of the reproductive stages and the plants took measures to eliminate the repression of *OsBIHD1*-induced BR catabolism. We speculate that the OsBIHD1-like protein WRKY45 relies on ubiquitination and proteasome protein degradation after fulfilling the task of immunity (Matsushita et al., 2013).

Future work will concentrate on the regulation of phosphorylation and ubiquitination of OsBIHD1 and to identify the transcriptional regulator of *OsBIHD1*. This study extends our knowledge concerning the mechanism whereby the *R* gene *Pik-H4* associates with *OsBIHD1* to balance growth and immunity involved in rice blast resistance.

## Author Contributions

YL, TG, and MH conceived the original screening and research plans. HW, SD, and GY supervised the experiments. HL, FG, and WX performed most of the experiments. ZC provided technical assistance to HL, JW designed the experiments and analyzed the data. WL conceived the project and wrote the article with contributions of all the authors. HL supervised and complemented the writing.

## Conflict of Interest Statement

The authors declare that the research was conducted in the absence of any commercial or financial relationships that could be construed as a potential conflict of interest.
