# Supplementary material for: NBS-LRR Protein Pik-H4 Interacts with OsBIHD1 to Balance Rice Blast Resistance and Growth by Coordinating Ethylene-Brassinosteroid Pathway
Source: Front Plant Sci. 2017 Feb 6;8:127. doi: 10.3389/fpls.2017.00127 (PMC5292422; doi:10.3389/fpls.2017.00127)
Supplement: Supplementary file 1 [file Presentation_1.PDF]

Title page

Title

NBS-LRR protein Pik-H4 Interacts with OsBIHD1 to Balance Rice Blast Resistance and Growth by coordinating Ethylene-Brassinosteroid pathway

Author names:

Hao Liu, Shuangyu Dong, FengweiGu, Wei Liu, Guili Yang, Ming Huang, Wuming Xiao, Yongzhu Liu, Tao Guo, Hui Wang, Zhiqiang Chen*, Jiafeng Wang*

Affiliations:

National Engineering Research Center of Plant Space Breeding, South China Agricultural University, Guangzhou, 510642, Guangdong, China.

***Co-corresponding author**:

Zhiqiang Chen,

E-mail: chenlin@scau.edu.cn

Address: National Engineering Research Center of Plant Space Breeding, South China Agricultural University, Guangzhou, 510642, Guangdong, China.

*** Corresponding author**

Jiafeng Wang,

E-mail: bcjfwang@gmail.com

Address: National Engineering Research Center of Plant Space Breeding, South China Agricultural University, Guangzhou, 510642, Guangdong, China.

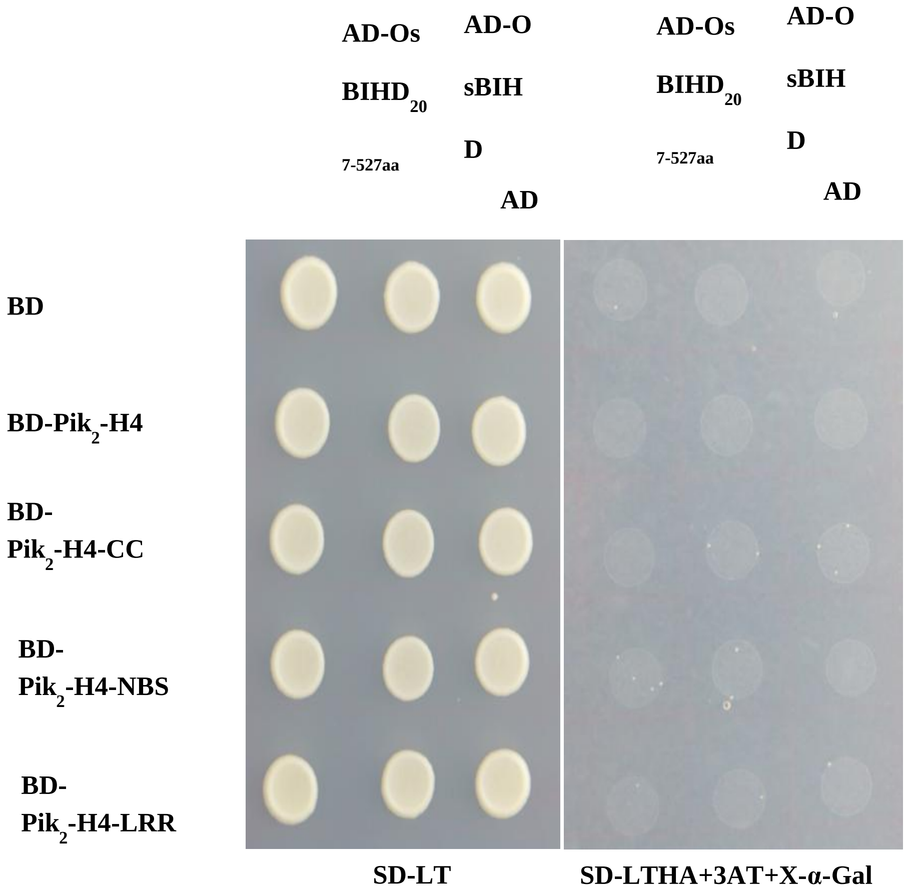

Figure S1.OsBIHD1 does not interact with Pik_2-H4 in yeast. The yeast cells were co-transformed with different combination of recombinant plasmid not grow on the SD-LTHA+3AT+X- α -Gal plate. These results indicate the OsBIHD1does not interact with Pik_2-H4 in yeast.

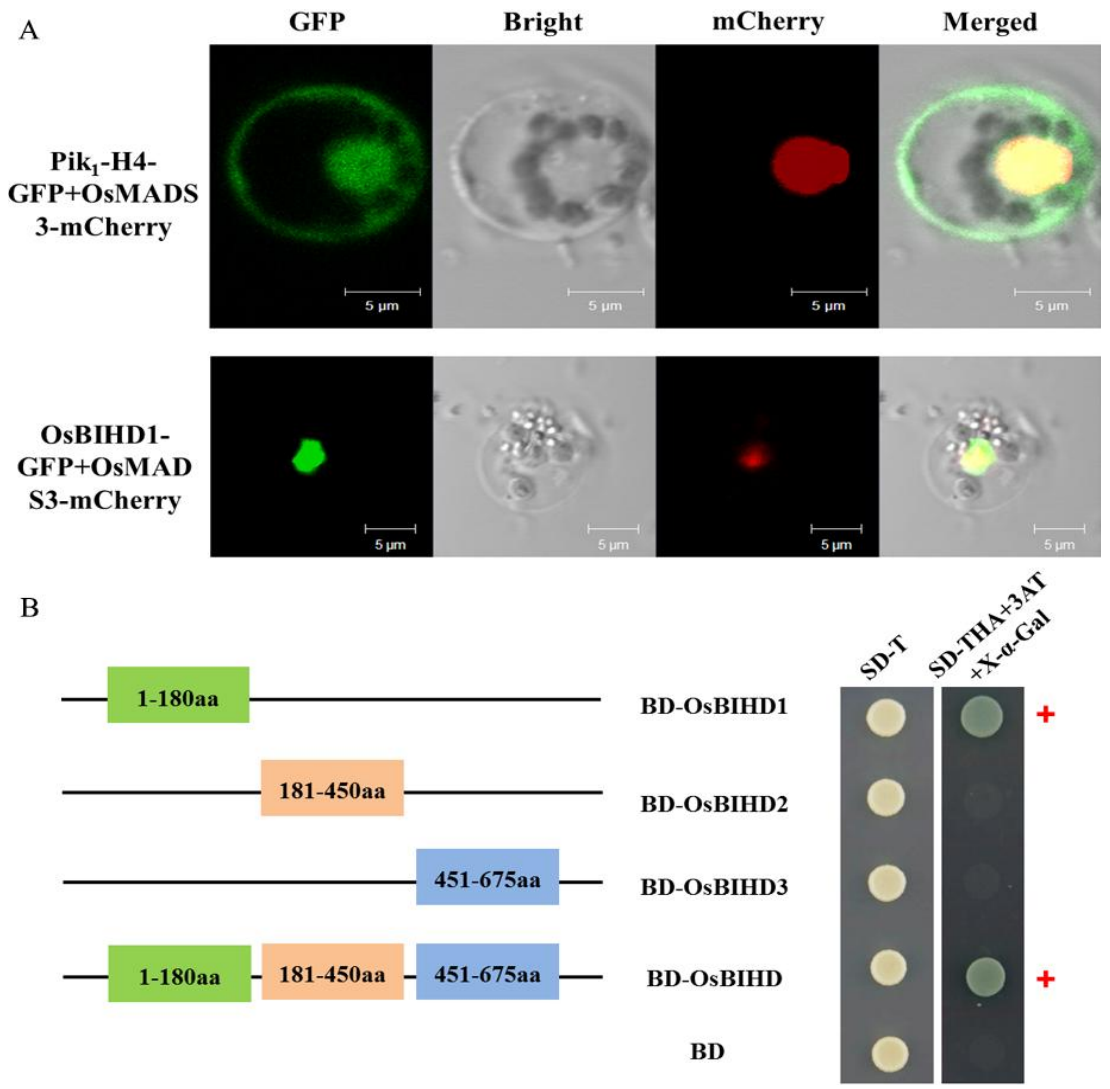

Figure S2. Subcellular localization and transcriptional activity assay of OsBIHD1. (A) Subcellular localization indicated that Pik1-H4 and OsBIHD1 co-localized in the nucleus. OsMADS3: mCherry was used as a nucleus marker. Pik1-H4-GFP and OsBIHD1-GFP signals are green, and the nuclear signals are red. Scale bar is 5 μm. (B) Transcriptional activation assay of OsBIHD1 and three truncated mutants fused with the GAL4 DNA-binding domain. Transcriptional activation activity was determined by the ability to activate the expression of the His, Ade and X-α-Gal reporter genes. BD-OsBIHD1 and BD-OsBIHDtransformants were selected on SD/-Trp/-His/-Ade with X-α-Gal and 100mM 3-AT plates. OsBIHD1 full length protein and its 1-180aa domain contained the transcriptional activity indicated by a positive (+) reaction in the X-α-Gal assay.

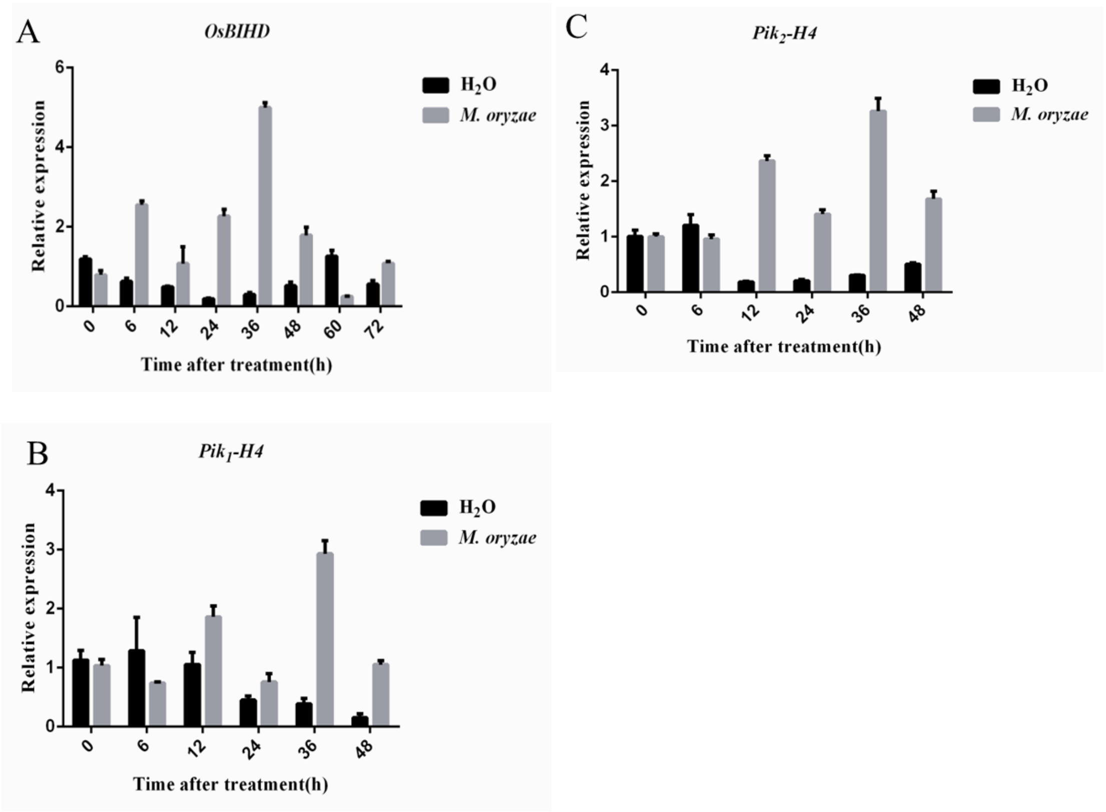

Figure S3.*OsBIHD1* and *Pik-H4* was up-regulated by *M. Oryzae*. A, Relative expression of *OsBIHD1* over a time course of 72 h after inoculation with *M.oryzae*GDYJ7 in wild-type. B-C, Relative expression of *Pik-H4* in wild-type after inoculated with *M.oryzae* in 48 h. The control group was treated by H_2O. The columns are means±SD of three biological replicates.

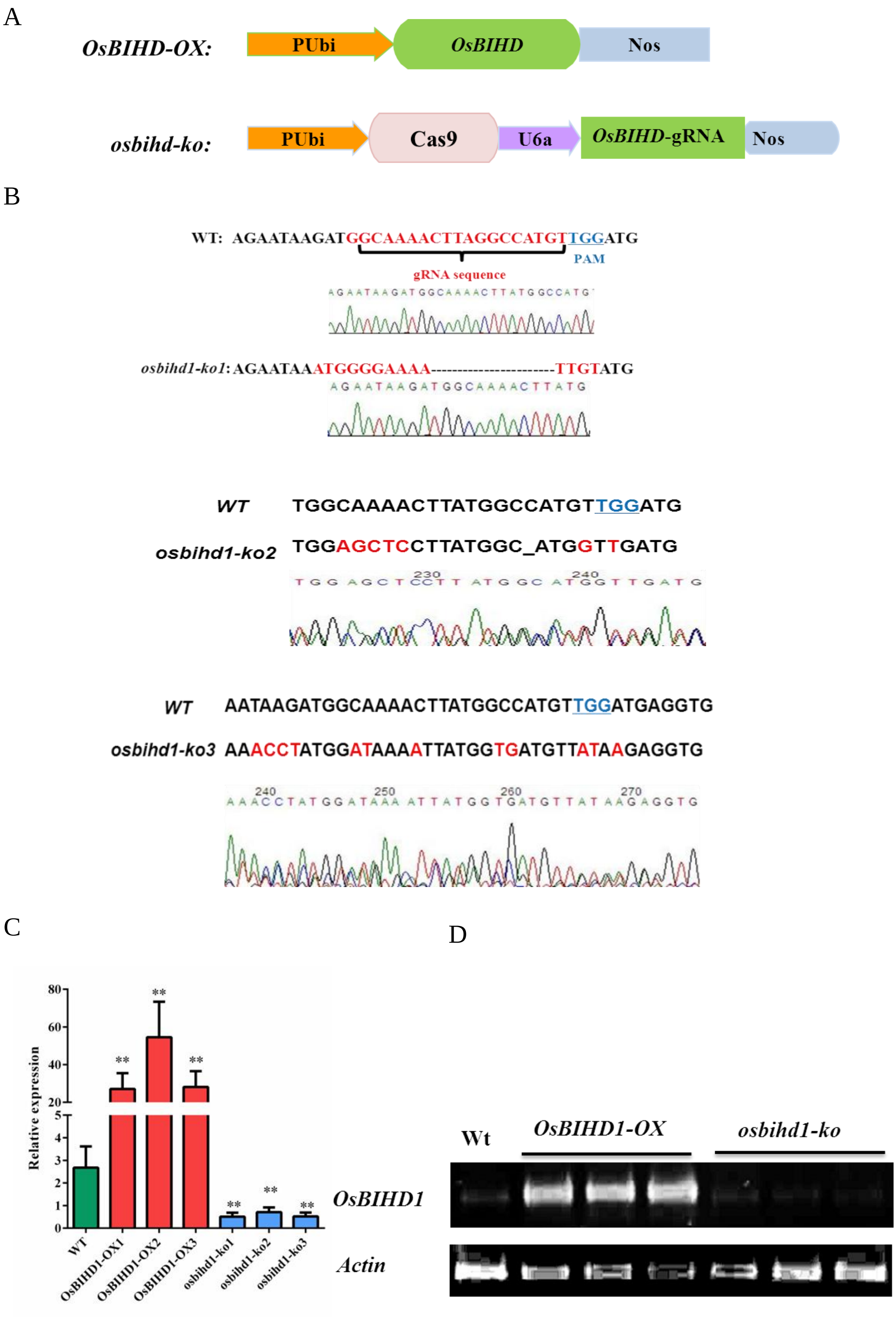
A
OsBIHD-OX:
PUbi
OsBIHD
Nos
osbihd-ko:
PUbi
Cas9
U6a
OsBIHD-gRNA
Nos
B
WT: AGAATAAGATGGCAAAACTTAGGCCATGTTGGATG
PAM
gRNA sequence
osbihd1-ko1: AGAATAAATGGGGAAAA----------------------TTGTATG
WT TGGCAAAACTTATGGCCATGTTGGATG
osbihd1-ko2 TGGAGCTCCTTATGGC_ATGGTTGATG
WT AATAAGATGGCAAAACTTATGGCCATGTTGGATGAGGTG
osbihd1-ko3 AAACCTATGGATAAAATTATGGTGATGTTATAAGAGGTG
C
D
Relative expression
80
60
40
20
5
4
3
2
1
0
**
WT
OsBIHD1-OX1
OsBIHD1-OX2
OsBIHD1-OX3
osbihd1-ko1
osbihd1-ko2
osbihd1-ko3
Wt
OsBIHD1-OX
osbihd1-ko
OsBIHD1
Actin

E

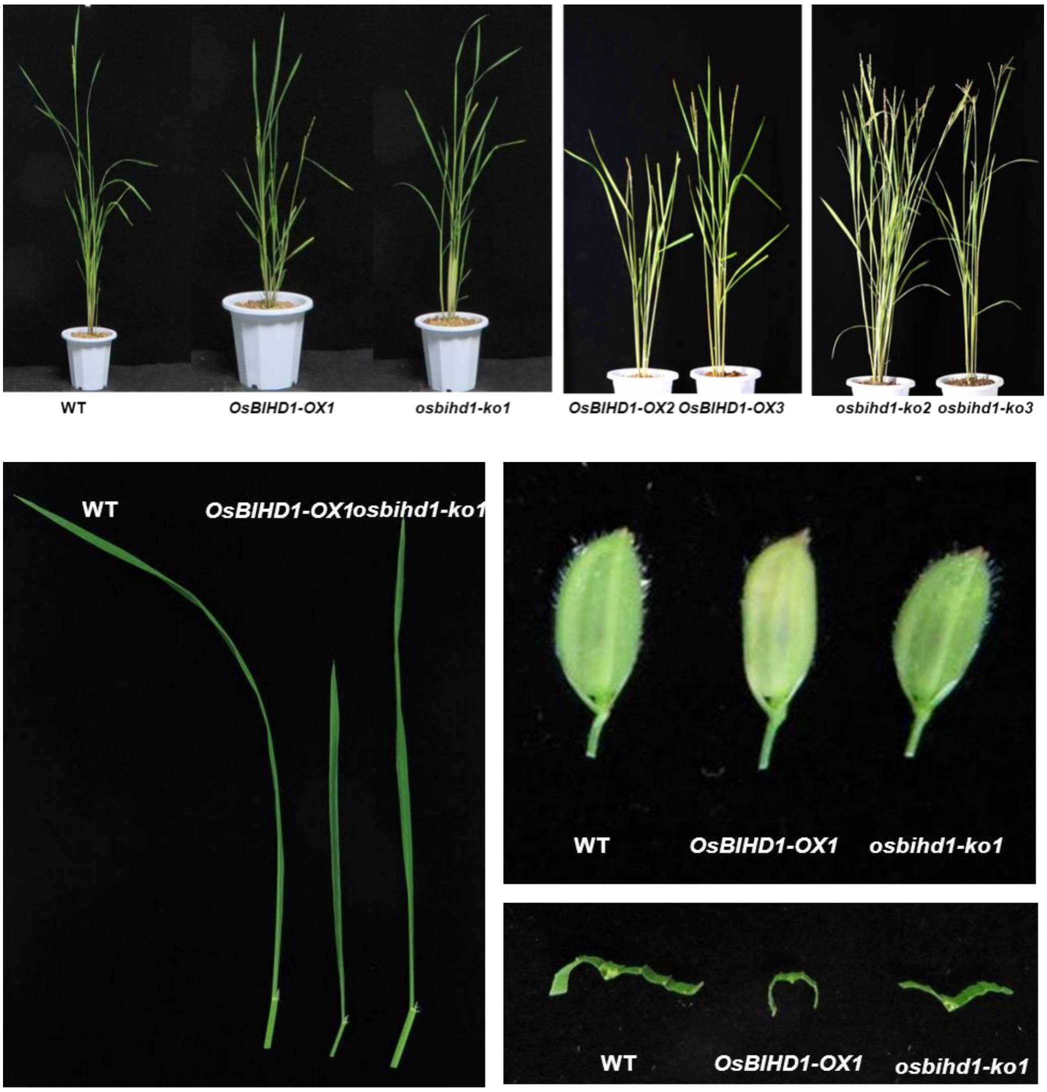

Figure S4.Identified the *OsBIHD1* transgenic plants in *Pik-H4* NILs background.A, vectors Schematic diagram of *OsBIHD1* transgenic plants. B, Target sequence of *OsBIHD1* CRISPR/Cas9 knock-out plant. Red bases is gRNA sequence, the blue bases indicated the PAM motif. C, Real-time PCR analysis of *OsBIHD1* expression in wild-type, *OsBIHD1-OX*, and *osbihd1-ko*. Values are means±SD of three biological replicates, and asterisks indicate a significant difference according to the t-test ($P < 0.05$) compared with wild-type. D, Semi-quantitative analysis of *OsBIHD1* expression in wild-type, *OsBIHD1-OX*, and *osbihd1-ko*. The Actin was used as endogenous reference. E，Phenotype of wild-type and *OsBIHD1* transgenic plants.

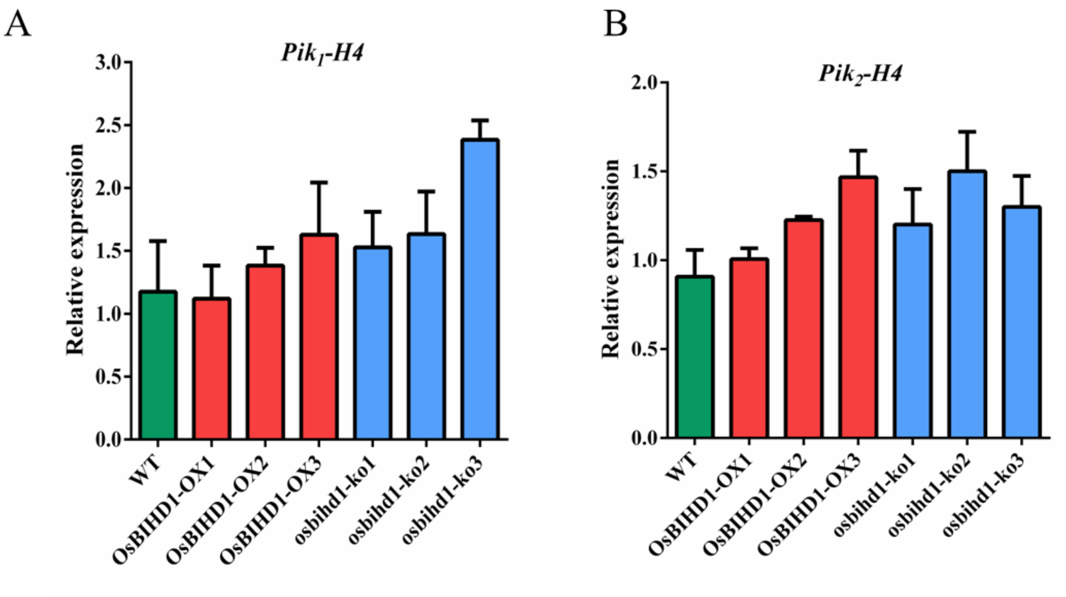

Figure S5. Relative expression of *Pik-H4* in *OsBIHD1* transgenic plants and wild-type under the normal growth condition. A, Relative expression level of *Pik*$_1$*-H4* in various *OsBIHD1* transgenic plants and wild-type. B, Transripts levels of *Pik*$_2$*-H4* in wild-type, *OsBIHD1-OX*, and *osbihd1-ko*, respectively. Values are means±SD of three biological replicates.

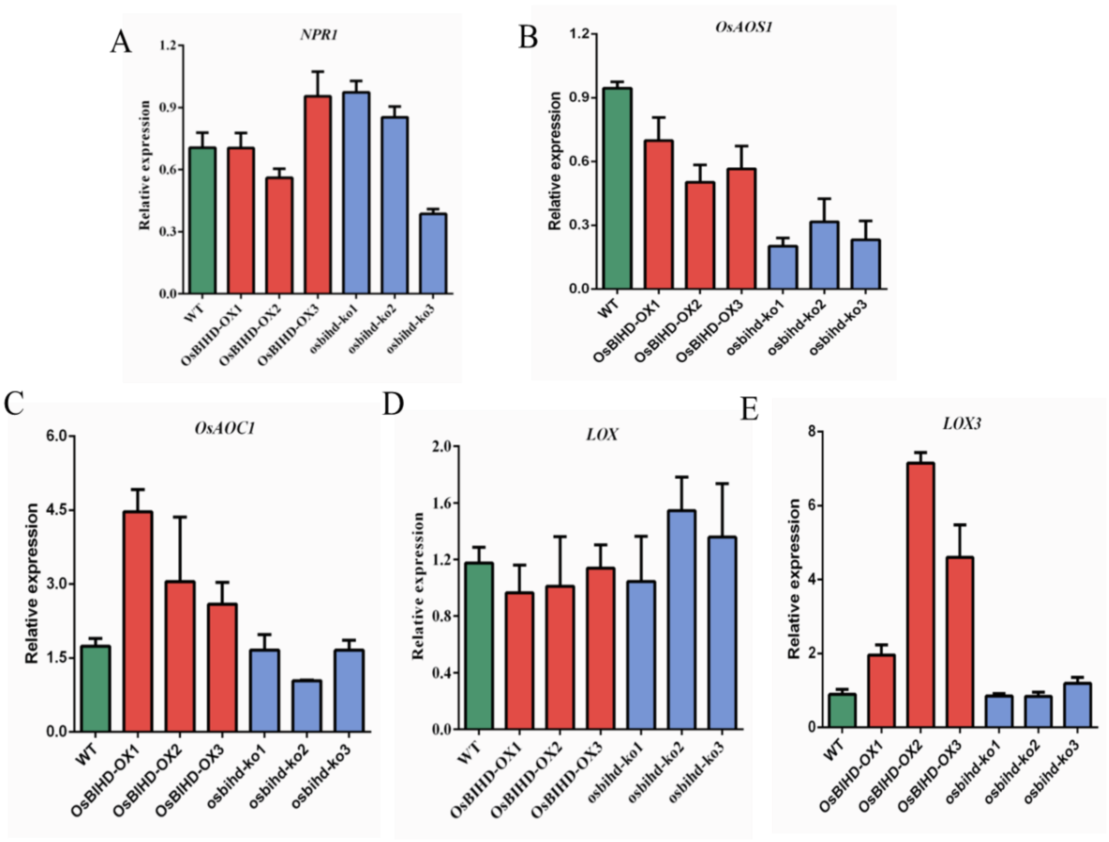

Figure S6. Relative expression of SA and JA biosynthetic genes in wild-type and *OsBIHD1* transgenic plants. A, Relative expression of *NPR1* in wild-type, *OsBIHD1-OX*, and *osbihd1-ko*.*NPR1* was not significantly changed in different plants. B-E, Relative expression of JA synthesis genes *OsAOS1*, *OsACO1*, *LOX*, and *LOX3* in wild-type, *OsBIHD1-OX*, and *osbihd1-ko*,respectively. Compared with wild-type, the transcript levels of *OsACO1* and *LOX3* obviously increased in *OsBIHD1-OX*. Values show aremeans±SD with three repeats.

A

Site	Sequence(5'-3')
Promoter(2Kb)	
-1596	ATTTGACAAATCAAATTGAT
-1545	TCTGTCATCATCAAATCCA
5'UTR sequence(1716bp)	
-1424	ACTGCACAGGTTGACAACG
-804	TACATTTGACAAATCAAATT
-853	AATTCTGTCATCATCAAATC

B

Site	Sequence(5'-3')
Promoter(2Kb)	
-1253	TCCCACCTGTCATACACAT
-547	TTTGACAAAAGAAAAGTTAA
-131	TGCATTGTCAGTTACTCCAG

Figure S7. PLACE database predicted the OsBIHD1 binding sites of *OsACO3* and *CYP734A2* promoter sequence. A, PLACE predicted the *cis*-acting regulatory DNA elements of OsBIHD1 in *OsACO3* promoter and 5'UTR sequence. The negative number indicated the sites that located in the upstream sequence of initiation codon ATG. Red bases suggested the conversed binding motif TGTCA/TGACA. B, PLACE predicted the OsBIHD1 binding sites in *CYP734A2* promoter region.

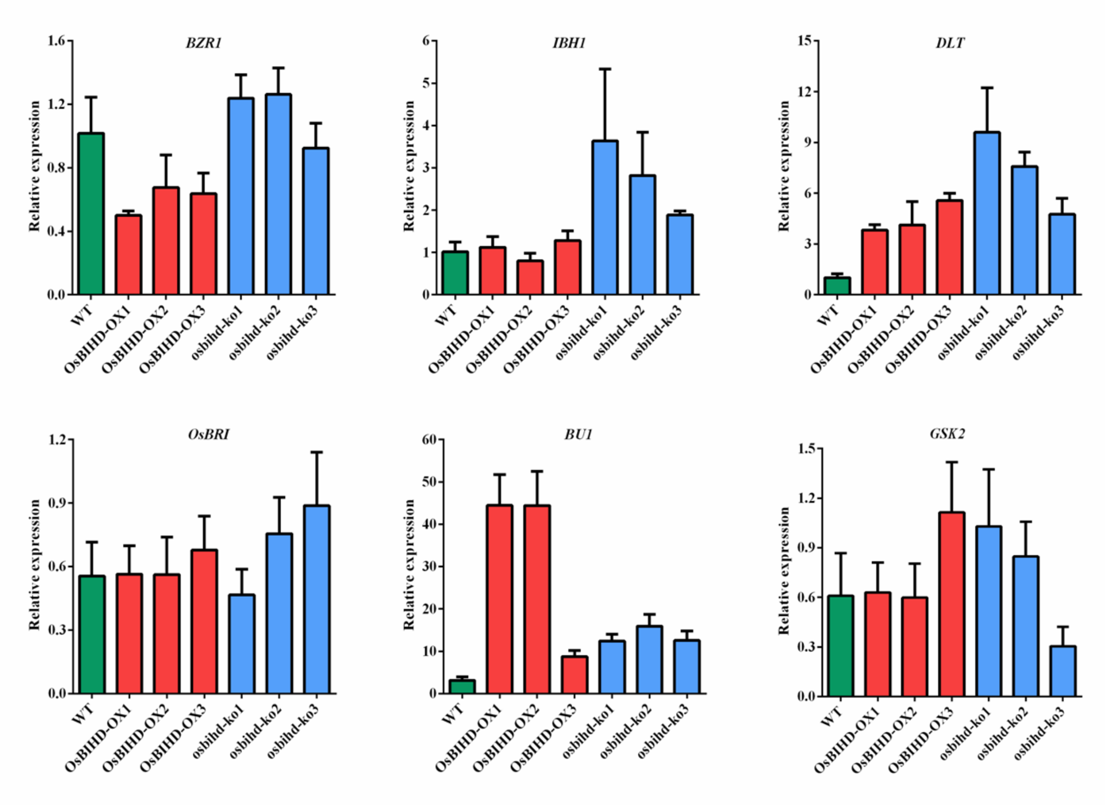

Figure S8. Relative expression of BR signaling genes in wild-type, *OsBIHD1-OX*, and *osbihd1-ko*, respectively. Values shown are means ±SD with three independent repeats.

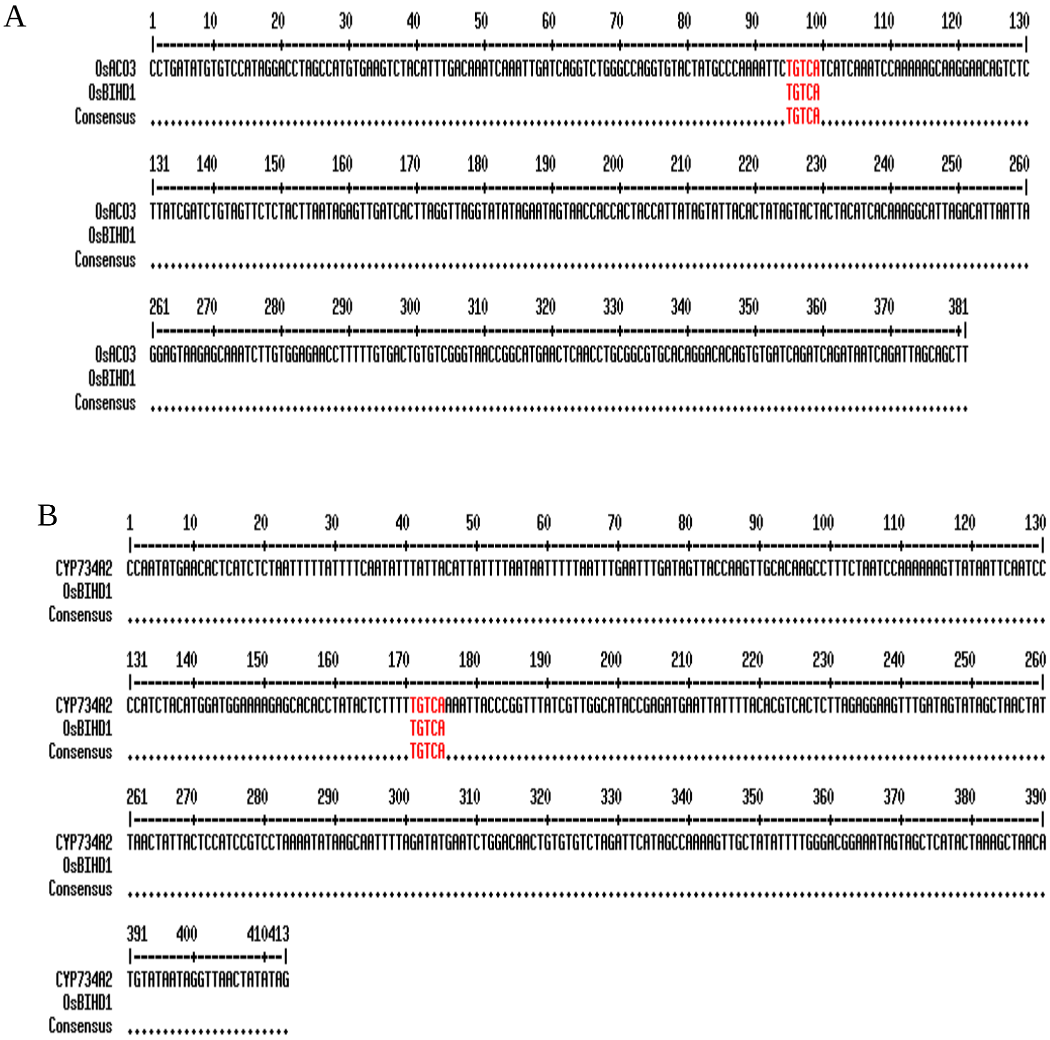

Figure S9.OsBIHD1 binding sites in the *OsACO3* and *CYP734A2* promoter region.

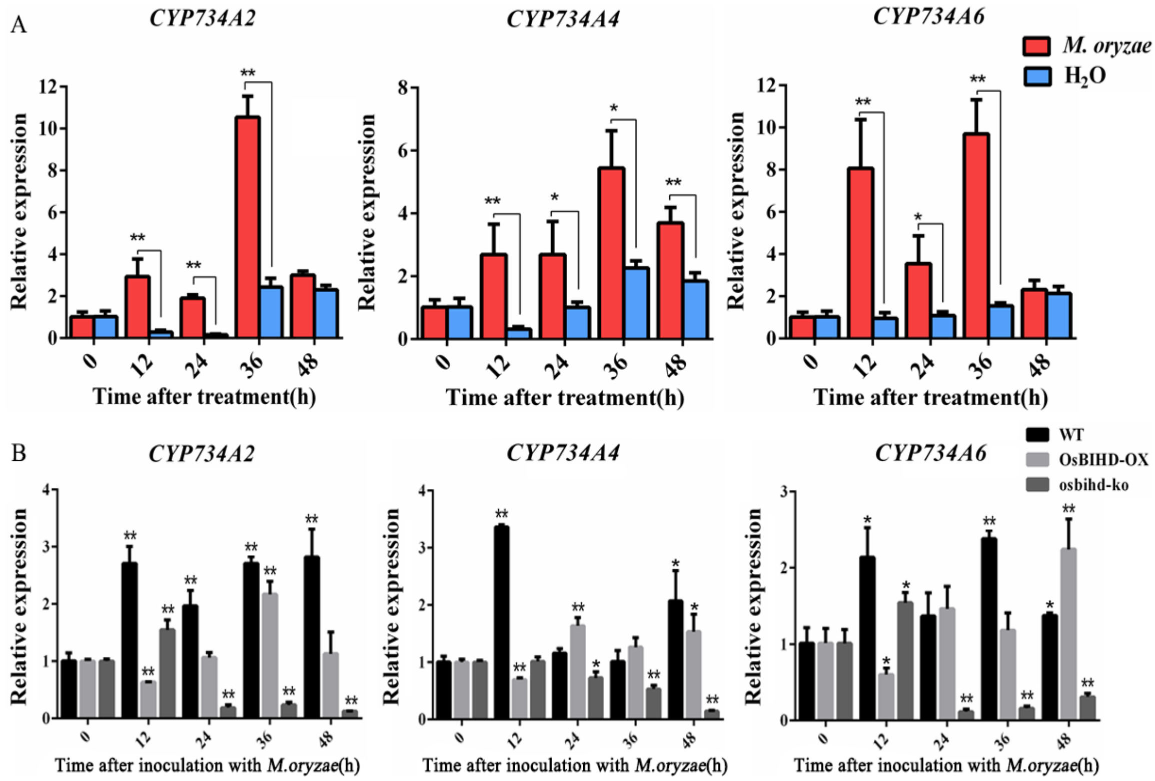

Figure S10. Transcript levels of BR catabolic genes are increased during an *M. oryzae* infection. (A) Relative expression of BR catabolic genes in wild-type, and asterisks indicate a significant difference according to the t-test ($P<0.05$) compared with control group. (B) Relative expression of BR catabolic genes in OsBIHD1-OX, and osbihd1-ko plants after inoculation with *M. oryzae* race GDYJ7. Values shown are means±SD with three independent repeats， and asterisks indicate a significant difference according to the t-test ($P<0.05$) compared with 0h.

Table S1. Primers used in various vectors construction

Primer name	Sequence	Restriction enzyme
**Y2H**		
Pik1-H4 F	gttgttCCCGGGTATGGAGGCGGCTGCCAT	*Sma*I
Pik1-H4 R	gttgttCCCGGGCTAGCTAGTAGTTTCTGTT	
Pik2-H4 F	gttgttCCCGGGTATGGAGTTGGTGGTAGGTGCT	
Pik2-H4 R	gttgttCCCGGGTCATGCAGTGACGATGCCATCA	
Pik1-H4 CC F	gttgttGAATTCATGGAGGCGCTGGCCATGGCCGTA	*EcoR*I
Pik1-H4 CC R	gttgttGGATCCCTAAAATTCACATATGGATTTCA	*BamH*I
Pik1-H4 NBS F	gttgttGAATTCCACAAGGTCAAAACAGTTTGCA	*EcoR*I
Pik1-H4 NBS R	gttgttGGATCCCTAGGAGGATGCACTAGTACTAG	*BamH*I
Pik1-H4 LRR F	gttgttGAATTCCCAAGGTTGATTCGCCGGCT	*EcoR*I
Pik1-H4 LRR R	gttgttGAATTCCTAGGCGCTGATGCCAGGGGCGT	
Pik1-H4 nLRR F	gttgttGAATTCACCATTGACGTCGTGAAGA	*EcoR*I
Pik1-H4 nLRR R	gttgttGGATCCCTAGCTAGTAGTTTCTGTTTG	*BamH*I
Pik2-H4 CC F	gttgttGAATTCATGGAGTTGGTGGTAGGTGCT	*EcoR*I
Pik2-H4 CC R	gttgttGGATCCTCACATCCCCACAGGCTCCTTTA	*BamH*I
Pik2-H4 NBS F	gttgttGAATTCAAGACGGTCATGGAGGAGTC	*EcoR*I
Pik2-H4 NBS R	gttgttGAATTCTCAGCCTTTTGTTGAACTTCCA	
Pik2-H4 LRR F	gttgttGGATCCATCTGAACTTGGCTCAAGTGAG	*BamH*I
Pik2-H4 LRR R	gttgttGGATCCTCATGCAGTGACGATGCCAT	
OsBIHD F	gttgttCATATGATGGCTACTTACTACTCGAGC	*Nde*I
OsBIHD R	gttgttGGATCCTCATCGAACCCACAGAGAAGC	*BamH*I
OsBIHD207-1581 F	gttgttCATATGCTTGATGAGGTCGTCAGTGTT	*Nde*I
OsBIHD207-1581 R	gttgttGGATCCGGTTCATGAAGCTGTCATCCT	*BamH*I
**BiFC**		
OsBIHD F	gttgttACCGGTATGGCTACTTACTACTCGAGC	*Age*I
OsBIHD R	gttgttACCGGTTCATCGAACCCACAGAGAAGC	
Pik1-H4 F	gttgttGCTAGCATGGAGGCGGCTGCCATGGCC	*Nhe*I
Pik1-H4 R	gttgttGCTAGCCTAGCTAGTAGTTTCTGTTTG	
**GST Pull-down**		
Pik1-H4 CC F	gttgttGGATCCATGGAGGCGGCTGCCATGGCC	*BamH*I
Pik1-H4 CC R	gttgttGAATTCAAATTCACATATGGATTTCA	*EcoR*I
OsBIHD207-1581 F	gttgttGGATCCCTTGATGAGGTCGTCAGTGTT	*BamH*I
OsBIHD207-1581 R	gttgttGGATCCGGTTCATGAAGCTGTCATCCT	
**Subcellular localization**		
OsBIHD F	gttgttTCTAGAATGGCTACTTACTACTCGAGCCCT	*Xba*I
OsBIHD R	gttgttGGATCCTCGAACCCACAGAGAAGCCATAGT	*BamH*I
Pik1-H4 CC F	gttgttGGATCCATGGAGGCGGCTGCCATGGCC	*BamH*I
Pik1-H4 CC R	gttgttCTGCAGGCTAGTAGTTTCTGTTTGAAT	*Pst*I
**Transcription activity assay**		

OsBIHD F	gttgttCATATGATGGCTACTTACTACTCGAGC	*Nde*I
OsBIHD R	gttgttGGATCCTCATCGAACCCACAGAGAAGC	*BamH*I
OsBIHD1-180 F	gttgttCATATGATGGCTACTTACTACTCGAGC	*Nde*I
OsBIHD1-180 R	gttgttGGATCCGTCATCACCATGGTAAGAGTG	*BamH*I
OsBIHD181-450 F	gttgttCATATGAACAGAATGAAGAATATGCAAT	*Nde*I
OsBIHD181-450 R	gttgttGATCCTTCTTTATACATGTCTTCGAT	*BamH*I
OsBIHD451-675 F	gttgttCATATGGAGATTGGGGAGGCGGATCTC	*Nde*I
OsBIHD451-675 R	gttgttGGATCCTCATCGAACCCACAGAGAAGC	*BamH*I
**Generate transgenic plant**		
OsBIHD-OX F	gttgttGGATCCATGGCTACTTACTACTCGAGC	*BamH*I
OsBIHD-OX R	gttgttGGATCCTCATCGAACCCACAGAGAAGC	
OsBIHD U6a F	gccGGGGGTATCTTCCAACCCAC	*Bsa*I
OsBIHD U6a R	aaacGTGGGTTGGAAGATACCCC	
Hpt-F	TCCGGAGCCTCCGCTCGAAGTAG	
Hpt-R	CTGAACTCACCGCGACGTCTGTC	
OsBIHDCasF-Test	CCAGGCTGAAGCAGGAAAATCAGA	
OsBIHDCasR-Test	CACTTCGATTTCATAGCGAGGTAT	
**Y1H**		
ACO3 F	gttgttGAATTCTTCAATTCATATTTTCTCGATCC	*EcoR*I
ACO3 R	gttgttACGCGTCTCTGTCTCTCTCTCTCGTTCAC	*Mlu*I
CYP734A2 F	gttgttGAATTCATAGGGATCGACAGCCTTGGATTT	*EcoR*I
CYP734A2 R	gttgttACGCGTCCTCCTCCTCCCCCCCTGTTTCTT	*Mlu*I
OsBIHD F	gttgttCATATGATGGCTACTTACTACTCGAGC	*Nde*I
OsBIHD R	gttgttGGATCCTCATCGAACCCACAGAGAAGC	*BamH*I
**GUS assay**		
Pcyp34a2-F	gttgttGGATCCCTATATAGTTAACCTAT	*BamH*I
Pcyp734a2-R	gttgttCCATGGCCAATATGAACACTCA	*Nco*I
Paco3-F	gttgttGGATCCCCTGATATGTGTCCATAGGA	*BamH*I
Paco3-R	gttgttCCATGGAAGCTGCTAATCTGATTATC	*Nco*I

Table S2. Primers used in RT-PCR

Primer name	Sequence
Pik1RT-F	AGCTGAATCATCTACAGACTCTG
Pik1RT-R	GAGGTCCGAAACGATCGATGATC
Pik2RT-F	ACTTGGCTCAAGTGAGATCACTG
Pik2RT-R	CAAGTACTCAAGTTTCTGAATCT
q-OsBIHD F	CTTACAGCATCCTGACAACAG
q-OsBIHD R	CTTTGATCTATTTGGTTCG
D2f	AgCTgCCTggCACTAggCTCTACAgATCAC
D2r	ATgTTgTCggAgATgAgCTCgTCggTgAgC
D11f	AgTgAAgAgggAgCATgAAggCA
D11r	ATCTgCAgggCTgAAATTgTTggg
DWAF-f	ATggTgTTggTggCgATTggggTggTTg
DWAF-r	ATgTTgTTCCgCCCCAggATgTCCAgCA
DWAF4-f	TTCATggAgCAgCACATCgC
DWAF4-r	CgggTAgCTgCACTCgAACA
BZR1f	CgTCgCCCACCTACAACCTC
BZR1r	TCgCCCAAATCgCAgCAT
IBH1f	CCgAACCCTAACCCTAgCgT
IBH1r	AggCCAgCATgTgCTTCgT
BU1f	AAgCTTTAgCTCCAgCCACC
BU1r	CCTgggCTgTTgTgATCCAT
BLEf	AggACggTgCTgTTCTCTTg
BLEr	gCAACAACATACATgggTTTTCC
BRIf	TCAgAACAACTACCTCACCggCg
BRIr	gCCggTTgCTCgCCAAA
RAV1f	ACCTCgATAACCCACATCCg
RAV1r	gAAggCAgCAgACggAAgAT
DLT-F	TgCggATACTCAACgCCATCA
DLT-R	ACTCgCCgACTCCggTgATC
GSK2f	CTGGTTCTTTCGGTATCGTCT
GSK2r	ATATTGGGTTCACCTGGGAC
CYP734A2f	ATGTGCGTAGGCCAGAACCT
CYP734A2r	GGTATAGGAGCATCAGCACTGTC
CYP734A4f	GACAGCGGCTTCAACGACTT
CYP734A4r	AGGTTGGTGGTCGTCTGCTT
CYP734A6f	GTTCATACCGTTCGGCCTTG
CYP734A6r	GACGTATCTGGCCGACAACC
LOX-F	GATACATATCTGCCTAGCA
LOX-R	AGGACTGGTCGTACTAGTT
ACO1-F	CTGCGGCGATGGAGCAGCTGGA
ACO1-R	CACGAACTTGGGGTACGCCACGA
ACS1-F	TCGGCCAAGACCCTCGACG
ACS1-R	CGAAAGGAATCTGCTACTGCTGC

UBQ-F	ACCCTGGCTGACTACAACATC
UBQ-R	AGTTGACAGCCCTAGGGTG
AOC-F	TACGAGATCAACGAGCGCGACC
AOC-R	TGTGGCCGTAGTCGCCGAAGTA
AOS1-F	TCTCCCACCGGCGGCAGCAGG
AOS1-R	GTCGGCGAGGCGGTCGTAGTC
LOX3-F	ACAAGAACTGGAACTTCACCGA
LOX3-R	AGCTCCGCGTCGCCTCGGAGCA
ACO2-F	AGCAACCCCGGCCTCGCTC
ACO2-R	AGGGACTTGCTATGACACGG
ACO3-F	AGCTGGAGGTGATCACCAAC
ACO3-R	TCGAACTTGTGGCGCACGTA
PR10-F	AAGTCGGATGTGCTCGAGG
PR10-R	GATGTCCTTCTCCTTCTCC
PR1a-F	AAGCTGGAGCACTCGGACT
PR1a-R	ACACCACCTGCGTGTAGTG
PR1b-F	ACTGGACGGCGGCGAGCGCG
PR1b-R	CTTATAGTTGCATGTGATG
NPR1-F	AGCTCGGATGACGGCACTC
NPR1-R	CTTCTGAAGAACATCCTGCA
2A Bs1-F	AGTTGTCCAGATTCATATCTA
2A Bs1-R	TAATTCAATCCCCATCTACAT
2A Bs2-F	TAATAGTTAATAGTTAGCTAT
2A Bs2-R	ATTTGATAGTTACCAAGTTG
2A Bs3-F	TCGCAGTGGATGGATTTTTG
2A Bs3-R	AGCCATAACCCGAGCTGA
ACO3 Bs2-F	ATAGGACCTAGCCATGTG
ACO3 Bs2-R	CTATAATGGTAGTGGTGGT
ACO3 Bs1-F	CCTGATATGTGTCCATAGGA
ACO3 Bs1-R	GTGGTGGTTACTATTCTATA
ACO3 Bs3-F	CTAGCCATGTGAAGTCTACAT
ACO3 Bs3-R	TAGTGTAATACTATAATGGTA
